# Graveyard Statistics of Warwick and Leamington

**Published:** 1857-04

**Authors:** John Webster


### GRAVE-YARD STATISTICS OF WARWICK AND LEAMINGTON.

By JOHN WEBSTER, M.D., F.R.S., etc.

HAVING lately occasion to visit Warwickshire, I occupied myself, during several leisure hours, with examining the gravestone records of Warwick and Leamington; thus carrying out, in these adjacent districts, inquiries analogous to those reported in previous numbers of this Review.

According to existing memorials in the churchyards of Warwick, from eighty to ninety years appear to be not uncommon ages; several exceeded that period; while one woman, buried in the St. Nicholas cemetery, had attained ninety-two at death. Contrasted with Warwick, the burying-grounds of Leamington indicated much lower ages; no individual buried in any cemetery in Leamington is named as having reached ninety years, as far as I could ascertain; eighty-eight being the highest figure reported on any grave-stone.

A more conclusive proof, shewing the inferior position occupied by Leamington in any longevity scale, may be adduced from its local registers of deaths, which were purposely inspected. At Warwick, twenty persons from eighty years and upwards—of whom one was ninety-one, and another ninety-two at death—have been interred, within the last eighteen months; at Leamington, only nine persons, who had lived to eighty and beyond that age, were similarly registered, the oldest being eighty-nine, a female. Possessing nearly double the population of Warwick, if long life prevailed amongst its inhabitants to the same extent, Leamington ought to have furnished about *forty* octogenarians, or persons even upwards of that advanced period; or, in other words, a quadruple proportion.

A feature illustrating the salubrity of Warwick, especially when it is contrasted with many other towns, is the small proportion of deaths recorded amongst its juvenile population; viz. those under five years of age. From an abstract of the parish registers of St. Nicholas, Warwick (with which I have been kindly favoured by the present incumbent), I find that amongst the inhabitants—who now range about 4,200—during the last fifteen years, the average proportion of young persons, under five years, buried there, was only 32 per cent out of 1396; whilst in many English towns, the ratio often reaches forty-five and fifty per hundred. Nay, this ratio is occasionally even greater; for in the neighbouring town of Coventry, during the last quarter, the registrar of the Holy Trinity district reported that, "out of the total of 156 deaths, 109 were of children under five years of age"; *i. e.* the high ratio of nearly 70 per cent. During the identical period of fifteen years, ending on the 31st of last December, seventy-one persons, whose ages varied from eighty to ninety, were interred, according to the St. Nicholas registers ; besides fourteen others, who are reported between ninety and ninety-nine at death. None, however, had actually become centenarians.

Another important fact in favour of Warwick is, that more aged individuals are now living in the above town than in Leamington ; notwithstanding the oldest living inhabitant of the Spa district, or Leamington, is a lady at present in her ninety-third year. Numerous cogent reasons might be easily adduced, explanatory of the differences just stated. Warwick stands on a rocky hill, having an acclivity, which, though somewhat abrupt, is not considerable, and the position otherwise appears favourable ; while, in several of its streets, there prevails free ventilation. Leamington, on the contrary, occupies a clayey soil, lies low in many places, and is near to the banks of a muddy, sluggish stream, which cannot but be insalubrious, notwithstanding several adjoining medicinal springs have become famous in public estimation, and however beneficial they may be in the alleviation or cure of various complaints. Further, its situation, particularly in the lower and most populous part, is somewhat damp ; while luxuriantly foliaged trees often grow rather close to numerous elegant, yet in many respects healthy villas, and even in wide, open streets. The above causes materially influence the salubrity of this town, and likewise the longevity of residents.

In conclusion, I would observe that sufficient proofs are here specified to warrant the inference that Warwick is a more salubrious and preferable residence than Leamington Priors ; although the latter is now frequently resorted to by invalids for the restoration of their health ; and by other persons, who select this favourite retreat, in order to enjoy comfort and tranquillity, during the evening of life, in an urban yet rural region ; which is not only rendered beautiful by its fertile and wooded landscapes, interspersed with country seats of resident gentry, but is in many respects historically interesting, more especially from its being in the native county of England's greatest bard, and human nature's truest delineator—the immortal Shakespeare.

24, Brook Street, March 1857.

